# Longitudinal Deficiency: A Case Report on Congenital Limb Deformity

**DOI:** 10.7759/cureus.30727

**Published:** 2022-10-26

**Authors:** Asna Tasleem, Alana Ebbitt E Ernharth, Elizabeth Imboden, Abdul Waheed

**Affiliations:** 1 Family and Community Medicine, WellSpan Good Samaritan Hospital, Lebanon, USA; 2 Pediatrics, Penn State College of Medicine, Hershey, USA; 3 Pediatric, WellSpan York Hospital, York, USA; 4 Family Medicine, Wellspan Good Samaritan Hospital, Lebanon, USA; 5 Family and Community Medicine, Penn State University College of Medicine, Milton S. Hershey Medical Center, Hershey, USA

**Keywords:** congenital limb reduction defect, ulnar longitudinal deficiency, vanishing, di-di twin gestation, limb deformity

## Abstract

Congenital limb reduction defects affect 1 in 1,900 babies born in the US. Limb reduction refers to the shortening or total absence of a limb or a specific segment of a limb. Congenital deficiencies of the upper limb are the most common location and comprise 58.5% of newborn limb reduction deficiencies. Longitudinal deficiencies affect the long axis of the limb and can affect one bone predominantly or can be hypoplasia or aplasia of several bones. To our knowledge, this case is a unique discussion of a baby born with longitudinal ulnar deficiency without any other commonly seen associated syndrome.

## Introduction

Congenital limb reduction defects affect 1 in 1,900 babies born in the US [1]. Limb reduction refers to the shortening or total absence of a limb or specific segment of a limb. Congenital defects of the upper limb are the most common form of limb reduction defects, as they constitute 58.5% of newborn limb reduction defects. [2] There are two broad categories of congenital limb defects, transverse, and longitudinal defects. In a transverse deficiency, all parts of a limb distally are absent and there is the preservation of all the segments proximal to the missing segment. [3] They are often caused by amniotic bands, which are fibrous bands of the amniotic sac that wrap the fetus as it develops. They can also be caused by Adams-Oliver syndrome, a rare hereditary disorder that is characterized by defects to limbs and the scalp. Deficiencies of the radius are the most common type of transverse deficiencies. These are often due to genetic causes or associated with other anomalies, such as Trisomy 18. Transverse deficiencies are more common than longitudinal deficiencies, which affect the long axis of a limb and can affect one bone predominantly. Hypoplasia or aplasia of several bones can also be seen in longitudinal deficiencies. Most longitudinal deficiencies are thought to be due to de novo mutations or due to exposure to teratogen in pregnancy. Thalidomide, a notorious teratogen used primarily during the 1950s-1960s for the treatment of nausea in pregnancy causes several longitudinal limb anomalies.

## Case presentation

**Figure 1:**
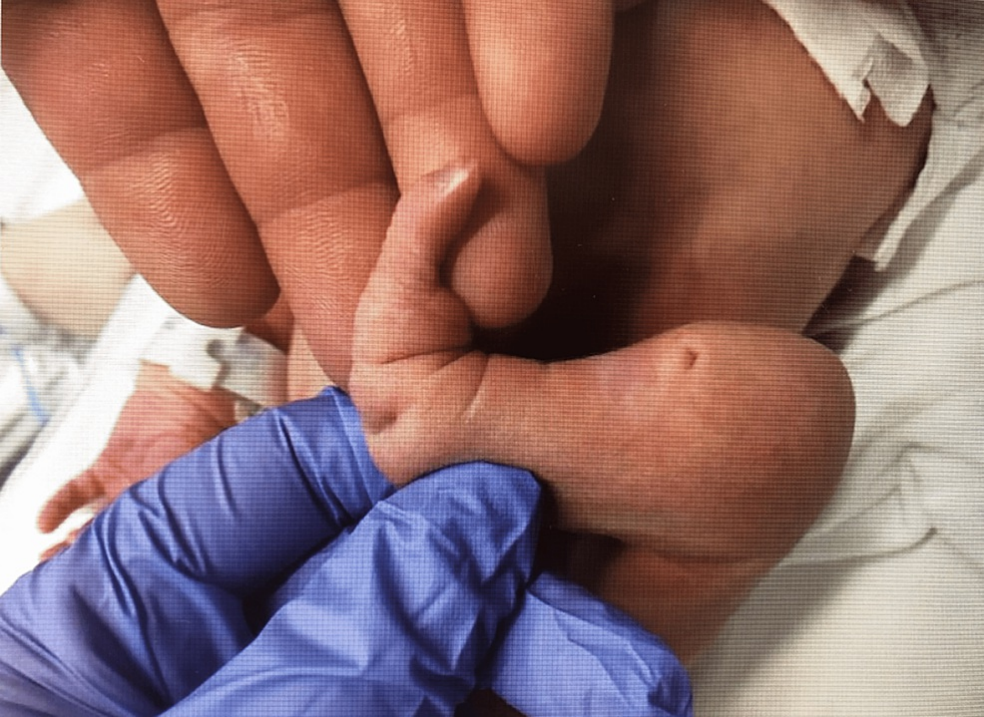
Skin defect visualized upon examination

The patient was a 37w5d female, born to a 32-year-old G4P2022 female via uncomplicated vaginal delivery. The patient was found to have a congenital anomaly in her upper right limb. She had a right-hand deformity with a single digit and a partial right ulna. There was also a small defect in the skin overlying the proximal portion of the partial radius (Figure 1).

**Figure 2:**
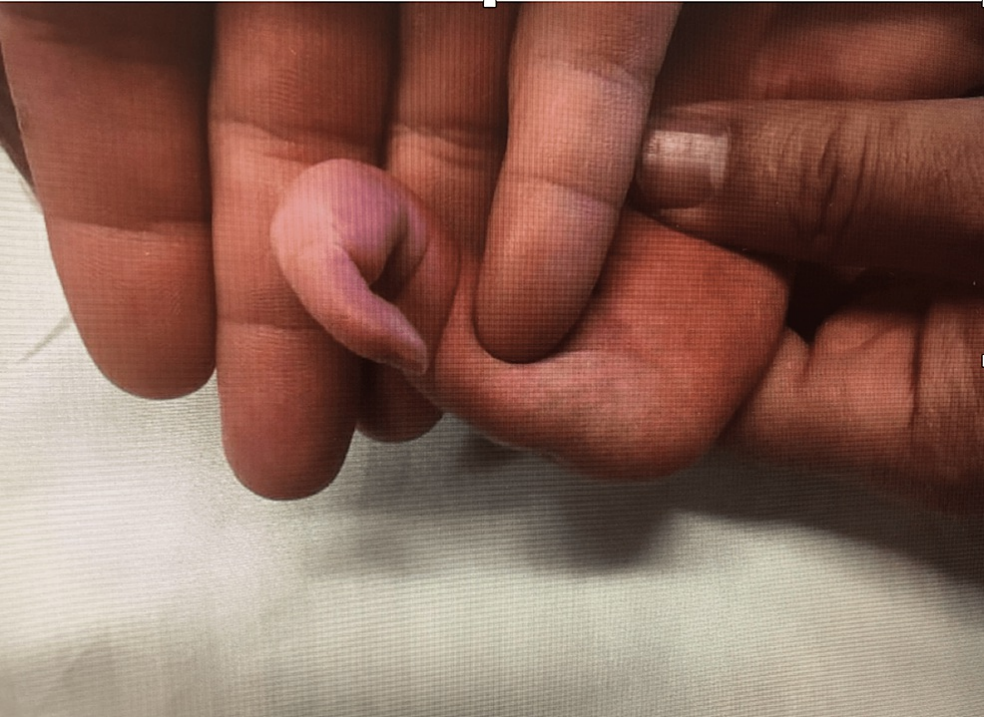
Limb abnormality at newborn day of life 2

Pregnancy was initially a dichorionic diamniotic twin pregnancy and other fetuses experienced a complication of vanishing twin syndrome. The pregnancy was also complicated by velamentous cord insertion, low pregnancy-associated plasma protein A (PAPP-A), and gestational hypertension. The patient's mother had received full prenatal care, including being followed by maternal-fetal medicine due to previous recurrent pregnancy loss. The mom was on aspirin, sertraline, and levothyroxine daily during the pregnancy and denied the use of tobacco, alcohol, or other drugs. The parents of the patient had no known genetic anomalies or family history of limb defects. The upper limb deformity was first visualized at the 20-week ultrasound, and during that time, the patient's parents did not choose to have any fetal genetic testing. Follow-up ultrasounds suggested that the ulna was severely shortened and only a single digit on the right hand was visualized. Besides the congenital limb deformity, the patient was also small for gestational age (2425g or 5lb 5.5oz) and had an incomplete cleft of the soft palate. She was otherwise healthy and without any other apparent limb deformities (Figure 2).

The patient had a successful range of motion of the elbow flexors, extensors, and wrist flexors and extensors. Capillary refill was less than 1 second when the digit was examined. Active digital flexion or extension was limited.

The patient passed her hearing and critical congenital heart disease screen, and the metabolic newborn screens were within normal limits. The patient was discharged home at two days of age with plans for close follow-up with the pediatrician, a cleft palate clinic, an orthopedic and plastic surgery team, along with whole exome testing; which the parents opted out of later. Imaging that was done in an outpatient setting concluded that this was an incompletely formed ulna with only the proximal one-third being present. The imaging suggested that this may represent an ulnar longitudinal deficiency.

Parents elected for observation until the patient started to crawl and did not request reconstruction surgery at that time. At subsequent visits, the patient failed the hearing test and had a risk of hearing loss. The patient had a family history of hearing loss, as her father was born with hearing loss in the right ear. Auditory brain stem response was evident for distortion product otoacoustic emission (DPOAE) measures being absent; consistent with abnormal cochlear outer hair cell functioning and/or middle ear functioning. Parents opted for cleft repair as well and the patient underwent furrow palatoplasty with tympanostomy tube placement successfully. The patient also underwent right pre-auricular remnant excision and tongue frenulectomy with no complications.

## Discussion

Ulnar longitudinal deficiency (ULD) is characterized by the lack of formation of the ulna, which frequently can lead to anomalies of the hand and fingers. One of the most common dysplasias of the ulna is the ulnar club hand, but it is still up to 10 times rarer than radial deficits [4]. The cause of ULD can sometimes be explained by inherited syndromes or other associated syndromes, but often, as is the likely case for this patient, the cause is unknown. One such inherited cause is Schinzel syndrome, which is a rare autosomal dominant genetic trait, which occurs as an abnormality on chromosome 12 (12q24.1) [5]. Schinzel syndrome is characterized by congenital anomalies of upper extremity bones along with hypoplasia and dysfunction of some apocrine glands and/or mammary glands. There are a variety of upper extremity limb anomalies that can occur in Schnizel syndrome, ranging from hypoplastic terminal phalanx to the complete absence of the ulna. An example of a non-inherited syndrome related to ULD is Cornelia de Lange syndrome, a multisystem disorder that includes growth and behavior deficits, dysmorphic facial features, and cardiac, gastrointestinal, and limb anomalies [6]. The majority of limb anomalies in Cornelia de Lange syndrome involve the ulna.

## Conclusions

Although this patient is not displaying characteristic findings of any syndromes, often ULD is associated with one. Therefore, proactive prenatal care to evaluate for ULD along with close follow-up and genetic testing is crucial for using the presence of ULD as a clue for potential other findings.
